# Pakistan Journal of Medical Sciences: A bibliometric assessment 2001-2010

**DOI:** 10.12669/pjms.333.13258

**Published:** 2017

**Authors:** Zameer Hussain Baladi, Loung V. Umedani

**Affiliations:** 1 Zameer Hussain Baladi, DPA. MLIS. M.Phil Lecturer – Cum – Librarian, King Saud Bin Abdulaziz University for Health Sciences, College of Applied Medical Sciences, Riyadh. Kingdom of Saudi Arabia; 2 Loung V. Umedani, MBBS, MPhil, Ph.D., Assistant Professor Basic Medical Sciences, College of Medicine, King Saud bin Abdulaziz University for Health Sciences, Riyadh, Kingdom of Saudi Arabia

**Keywords:** Pakistan journal of medical science, Pakistan health research council, Bibliometrics, Electronic libraries

## Abstract

**Objective:**

The aim of this study was to measure the growth of scientific research, authors’ productivity, affiliation with the institute and geographic locations published in the Pakistan Journal of Medical Sciences during the period of 2001 – 2010.

**Methods:**

This numerical analysis was conducted during mid-August 2016 to mid-October, 2016. The data for the study was downloaded from websites of e-journal of Pakistan Journal of Medical Sciences (PJMS) and Pak Medi-Net Com.

**Results:**

A total number of 1199 articled were covered by PJMS in 10 volumes and 40 issues with contribution of 3798 (3%) authors during 2001 – 2010. The average number of papers per issue is 30%. A gender wise contribution of males was higher 3050 (80%) than the females 748 (20%). A majority of articles were multi-authored 1052 (87%) as opposed to single author contribution 147 (13%). All 1199 articles were covered under four major disciplines i.e Basic medical sciences, medicine & allied, surgery & allied and radiological sciences and 39 sub-specialties according to medical subject headings (MeSH). It observed that 467 (39%) articles were published in Pakistan and 732 (61%) articles produced by other 32 countries. The Karachi city of Pakistan has produced 199 (16%) articles as highest as its national level and followed by Tehran (Iran) 77 (6%) as followed internationally.

**Conclusion:**

This study reveals that the participation of 32 countries in the PJMS publications proves it to be an internationally circulated journal to support research with the constant approach of publishing articles to each volume in basic medical sciences, biomedical, clinical and public health sciences.

***Abbreviations:***

DOAJ: Directory of Open Access Journals

IMEMR: Index Medicus Eastern Mediterranean Region

HEC: Higher Education Commission (Pakistan)

PJMS: Pakistan Journal of Medical Sciences

MeSH: Medical Subject Headings

PMDC: Pakistan Medical & Dental Council

SCIE: Science Citation Index Expanded

## INTRODUCTION

Information science is concerned with the collection of information for storage to classify into multidiscipline and retrieval. Bibliometric is a branch of information science, it identifies the numerical places of research productivity, and it quantifies to evaluate the publication and pattern of authorship. The bibliometric study is widely used for mapping of scientific research growth, authorship pattern, research collaboration, author’s productivity in any discipline of knowledge. It is mainly the best instrument in social science research for systematic analysis of publication output of any subject.^1^ Ibrahim M, Jan SU analyzed the publications of original articles in the Journal of Pakistan Medical Association from 2009 – 2013, they stated that a total number of 913 original articles were found in regular issues of the JPMA. The number of articles increased steadily from 148 to 214 respectively from 2009 to 2013. Three author contributions ranked the highest with 206 articles; 481 authors were geographically affiliated to Province of Sindh, Pakistan.^2^

Quinn N. et al. provided a historical perspective of publications in the discipline of pediatric, they say that Pediatrics as a specialized field of medicine only developed in the mid- 19th century. Archives of Pediatrics was the first pediatric journal and was established in 1884. Since then the number of publications on the subject of pediatric has dramatically expanded. There are now 191 journals solely dedicated to the specialty of pediatrics and published 497,240 articles during 1945 to 2010 among in these journals.^3^ Journals play an important role in the scholarly communication of different domain from very past by containing the original thought contents, ideas, views, research works and findings of researchers, scholars and academicians. Citation data can be used in many ways for a variety of purposes. In a competitive environment, it is important for an institution to show how performance supports its mission.^4^ Mergio JM, and Nunez A, reported that a bibliometric study was conducted under the title of Influential journals in health research: a bibliometric study; to identify the leading journals over the last 25 years (1990–2014) on Public Health, Environmental and Occupational Health, Health Management and Economics, Health Promotion and Health Behavior, Epidemiology, Health Policy and Services, Medicine, Health Informatics, Engineering and Technology, and Primary Care. The results indicate a wide dispersion between categories being the American Journal of Epidemiology, Environmental Health Perspectives, American Journal of Public Health, and Social Science & Medicine, the journals that have received the highest number of citations over the last 25 years.^5^ Bibliometric studies have been conducted on journals related mainly to scientific fields and are based principally on various metadata elements such as author, title, subject, citations and so forth. This type of analysis provides useful indicators of trends, scientific productivity, emphasis on research in various fields, and researcher preferences for publication. Typically, bibliometrics consider organization, classification, and quantitative evaluation of publication patterns as well as provide an analysis of macro-communication.^6^ Authorship studies describe author characteristics and authorship of articles and degree of collaboration of a specific group of authors.^7^

In the present era of information explosion, research in science and technology is rapidly progressing not only in the area of pure sciences domain but also in applied sciences. Scientists are increasingly working in collaboration in order to gain their expertise in areas of their specialization. Today research has become interdisciplinary and scientists in one area have to collaborate with scientists in other areas in order to fulfill the goals of research as per objectives. They realize the necessity of collaboration in research to make it useful for human welfare.^8^

The Pakistan Journal of Medical Sciences is a bi-monthly, peer-reviewed journal for medical & allied medical community. It accepts original research, review articles, case reports, guest editorials, short communications, drug trials and letters to editors. It is recognized by the Higher Education Commission (HEC), Government of Pakistan. It is indexed and abstracted in Science Citation Index Expanded (SCIE) by Thompson/ISI USA, Web of Science, Embase/Excerpta Medica Netherlands, WHO IMEMR Current Contents, Index Copernicus Poland, SCOPUS, INIS Database, CAB Abstract and Global Health UK, Pakistan Science Abstract, Registered with International Serials Data System of France, approved by the Pakistan Medical & Dental Council, Islamabad and covered by Pakmedinet, Open-J gate India, DOAJ and emrmedex.com.^9^ Currently it is also covered by PubMed Central since January 2013.

## METHODS

This is retrospective study, the objectives were set to explore for study 1) the growth of publication in all volumes & issues; 2) evaluate the contribution of authors in publications with gender wise distribution of the first author; 3) investigate the classification of published articles by medical specialties; 4) categorize the pattern of authorship; 5) identify the most contributed cities of Pakistan; and 6) the geographic affiliation of authors. For answering the above objectives, the data of original articles, review articles and case reports published in PJMS during the year 2001 to 2010, was collected & downloaded for statistical analysis in MS Office (Excel 2010) in the library of College of Applied Medical Sciences, King Saud bin Abdulaziz University for Health Sciences Riyadh, Kingdome of Saudi Arabia throughout the period of mid-August 2016 to mid-January 2017 from websites of the respective e-journal of Pakistan Journal of Medical Sciences (PJMS) http://www.pjms.com.pk/index.php/pjms and from Pak Medi-Net http://www.pakmedinet.com/ for a participatory research work for the references related to medical research and biostatistics.

## RESULTS

The PJMS published a total of 1199 papers in forty issues during the years 2001-2010 and 29.9% articles published per issue. During the period, the volumes increased progressively from 17 to 26 and there was also progressive year wise increased in the number of publications from 2001 to 2010 (Table-I). The annual growth rate of publication was max. 59.8% in 2007 and min. 3.5% in 2010.

**Table-I:** Distribution of articles Year, Volume & Issue-Wise from 2001 – 2010.

*Year*	*Vol:*	*Issues I*	*Issues II*	*Issues III*	*Issues IV*	*Total articles with %*	*Average articles per issue*	*Annual Growth Rate*
2001	17	7	10	8	13	38	9.5	
2002	18	9	14	14	13	50	12.5	31.5%
2003	19	11	16	15	15	57	14.25	14 %
2004	20	16	17	19	22	74	18.5	29.8%
2005	21	21	23	26	26	96	24	29.7%
2006	22	21	28	30	33	112	28	16.6%
2007	23	36	37	37	69	179	44.75	59.8%
2008	24	39	67	33	49	188	47	5%
2009	25	32	68	35	64	199	49.75	5.8%
2010	26	49	51	49	57	206	51.5	3.5%
Articles published in all issues	241	331	266	361	1199	119.9		

**Fig.1:**
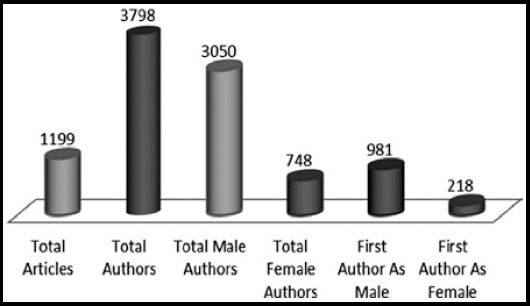
Authors contribution with gender specification in PJMS from 2001-2010.

The contribution of authors for the 1199 articles published in PMJS during 2001– 2010 was analyzed. The total number of authors who contributed to articles was 3798. The average number of authors per article was found 3.20. It also revealed that 3050 (80.3%) of the authors were male and 748 (19.6%) were females for the 1199 articles (Table-II). The males as the first author were 981 (81.8%) versus 218 (18.1%) females (Fig.1).

**Table-II:** Authors contribution with gender specification in PJMS from 2001 – 2010.

*Total Articles in PJMS 2001 – 2010.*	*2001*	*2002*	*2003*	*2004*	*2005*	*2006*	*2007*	*2008*	*2009*	*2010*	*Authors per article %*
	38	50	57	74	96	112	179	156	231	206	1199
Total Authors	101	132	189	223	305	312	553	604	717	662	3798 (3.16%)
Total Male Authors	78	92	150	185	67	268	436	493	568	509	3050 (80.30%)
Total Female Author	23	40	39	38	13	44	117	126	149	153	748 (19.69%)
Male as First Author	29	33	45	60	20	100	145	166	148	161	981 (81.81%)
Female as First Author	9	17	12	14	6	12	34	23	37	45	218 (18.18%)

**Fig.2:**
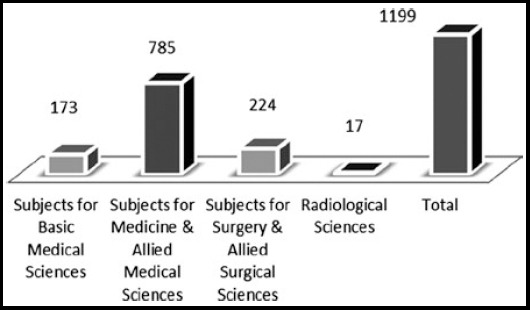
Classification of published articles by the major medical specialties in PJMS from 2001-2010.

Fig.2 shows a major contribution of publications relate to the major disciplines e. g Basic medical sciences, medicine & allied medical, surgery & allied surgical and radiological sciences.

Table-III shows a breakdown of the subjects. It is revealed that a total of 39 sub-specialties were covered by PJMS in 2001 – 2010.

**Table-III:** Classification of published articles by medical specialties in PJMS from 2001-2010.

*S. No*	*Classification of published articles by medical specialties*	*Article Published*	*%*
1	Community Medicine	94	7.84%
2	Oncology	87	7.26%
3	General Medicine	78	6.51%
4	Psychiatry	84	7.01%
5	Hematology	63	5.25%
6	Surgery	61	5.09%
7	Endocrinology	59	4.92%
8	Immunology	57	4.75%
9	Cardiology	53	4.42%
10	Pediatrics	44	3.67%
11	Urology	42	3.50%
12	Gynecology	35	2.92%
13	Infectious Diseases	33	2.75%
14	Obstetrics	33	2.75%
15	Pulmonology	32	2.67%
16	Orthopedics	31	2.59%
17	General Medicine	30	2.50%
18	Hepatology	30	2.50%
19	Dermatology	27	2.25%
20	Neurology	27	2.25%
21	Clinical laboratory science	23	1.92%
22	Medical Education	22	1.83%
23	Gastroenterology	21	1.75%
24	Bacteriology	20	1.67%
25	Pharmacology	19	1.58%
26	Radiology	17	1.42%
27	Otorhinolaryngology	12	1.00%
28	Ophthalmology	10	0.83%
29	Epidemiology	7	0.58%
30	Anesthesia	6	0.50%
31	Emergency Medicine	6	0.50%
32	Anatomy	5	0.42%
33	Biochemistry	5	0.42%
34	Nursing	5	0.42%
35	Toxicology	5	0.42%
36	Nutrition	4	0.33%
37	Occupational Medicine	4	0.33%
38	Hospital Management	3	0.25%
39	Microbiology	3	0.25%
40	Medical Jurisprudence	2	0.17%
		1199	

**Fig.3:**
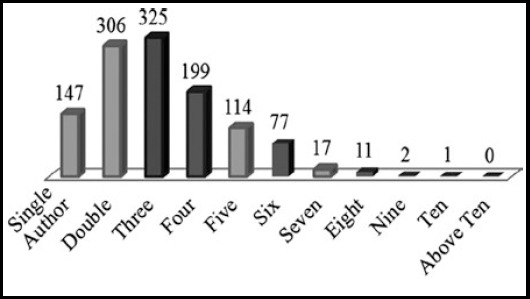
Authorship Pattern in PJMS from 2001-2010.

Fig.3 shows that PMJS published 1199 articles in forty issues during 2001 - 2010, among these articles 147 (12.2%) articles were written solos; and 1052 (87.7%) articles were written by multi authors. A breakdown of the authorship pattern indicated in Table-IV.

**Table-IV:** Author ship pattern in all issues in PJMS 2001 – 2010.

*Authorship Pattern*	*All Issues 2001*	*All Issues 2002*	*All Issues 2003*	*All Issues 2004*	*All Issues 2005*	*All Issues 2006*	*All Issues 2007*	*All Issues 2008*	*All Issues 2009*	*All Issues 2010*	*No of articles written by Authors*
Single Author	7	10	10	17	12	24	25	13	13	16	147 (12.2%)
Two Authors	13	14	9	17	29	28	49	60	33	54	306 (25.5%)
Three Authors	9	17	13	12	18	30	42	53	61	70	325 (27.1%)
Four Authors	5	6	10	17	16	21	34	25	40	25	199 (16.6%)
Five Authors	3	1	8	4	11	4	14	19	26	24	114 (9.5%)
Six Authors	1	1	7	2	8	2	10	13	20	13	77 (6.4%)
Seven Authors	0	0	0	4	2	1	3	2	3	2	17 (1.4%)
Eight Authors	0	1	0	1	0	1	1	2	3	2	11 (0.9%)
Nine Authors	0	0	0	0	0	0	1	1	0	0	2 (0.17%)
Ten Authors	0	0	0	0	0	1	0	0	0	0	1 (0.08%)
Above Ten Authors	0	0	0	0	0	0	0	0	0	0	0
Contribution of Multi Authors	38	50	57	74	96	112	179	188	199	206	1199
	3.17%	4.17%	4.75%	6.17%	8.01%	9.34%	14.93%	15.68%	16.60%	17.18%	

**Fig.4:**
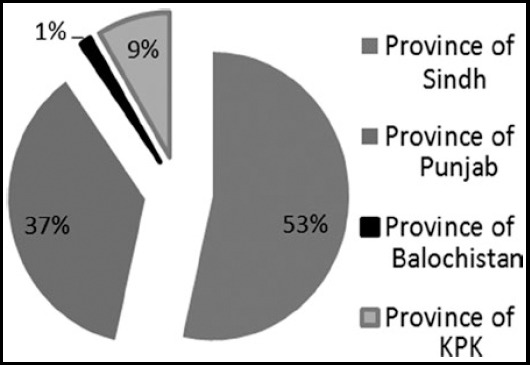
Province wise affiliation of authors in PJMS from 2001-2010.

Fig.4 shows the Province wise affiliation of authors, Province of Sindh 250 (53%), Punjab 171 (37%), KPK 40 (9%) and respectively Balochistan with 6 (1%).

Table-V highlights breakdown of city wise affiliation of authors in contribution of 467 (38.9%) out of 1199 articles, related to twenty-six cities of Pakistan published in PJMS 2001 – 201. The only city of Karachi Pakistan produced majority of 199 (16.60) articles, and city of Lahore produced 90 article respectively.

**Table-V:** City wise affiliation of authors in PMJS 2001 – 2010 (Largest to Smallest).

*S. No*	*Table 5: City wise affiliation of authors in PMJS* *2001 – 2010 (Largest to Smallest)*	*Total Published Articles*
1	Karachi	199 (16.60%)
2	Lahore	90 (7.51%)
3	Islamabad	30 (2.50%)
4	Rawalpindi	27 (2.25%)
5	Peshawar	24 (2.00%)
6	Hyderabad	18 (1.50%)
7	Jamshoro	15 (1.25%)
8	Faisalabad	11 (0.92%)
9	Mirpurkhas	10 (0.83%)
10	Abbottabad	7 (0.58%)
11	Multan	7 (0.58%)
12	Quetta	6 (0.50%)
13	Bahawalpur	4 (0.33%)
14	Nawabshah	4 (0.33%)
15	Attok	3 (0.25%)
16	Swat	2 (0.17%)
17	Bhakar	1 (0.08%)
18	DGK	1 (0.08%)
19	DIK	1 (0.08%)
20	Hazara	1 (0.08%)
21	Jacobabad	1 (0.08%)
22	Khairpur	1 (0.08%)
23	Larkana	1 (0.08%)
24	Mansehar	1 (0.08%)
25	Sukkur	1 (0.08%)
26	Toba Tek Singh	1 (0.08%)
		467 (38.95%)

Table-V shows that 732 (61.05) articles related to thirty-two geographic locations of authors, Islamic Republic of Iran contributes with 355 (29.61) articles in publication of PJMS 2001 - 2010, Turkey 85 (7.09), Nigeria 61 (5.09), Kingdome of Saudi Arabia 57 (4.75), Jordan 33 (2.75), Bangladesh 27 (2.25), India 24 (2.00), United Kingdome 12 (1.00), Malaysia 9 (0.09), Iraq & United Arab Emirates 8(0.67), Kuwait & Palestine 7 (0.58), Oman 5 (0.42), Canada 4 (0.33), Nepal, Poland, South Korea, Thailand & Tunisia produced 3 (0.25), Ireland, South Africa & United States of America 2 (0.17), Afghanistan, Bahrein, Cameroon, China, Greece, Russia, Spain, Sudan & West Indies contributed with 1 (0.08) articles from 1199 articles.

**Table-VI:** The geographic affiliation of authors in PMJS 2001 – 2010 (Largest to smallest).

*S. No*	*The geographic affiliation of Authors in PMJS 2001 – 2010 (Largest to Smallest)*	*Published Articles*
1	Iran	355 (29.61%)
2	Turkey	85 (7.09%)
3	Nigeria	61 (5.09%)
4	Saudi Arabia	57 (4.75%)
5	Jordan	33 (2.75%)
6	Bangladesh	27 (2.25%)
7	India	24 (2.00%)
8	United Kingdome	12 (1.00%)
9	Malaysia	9 (0.75%)
10	Iraq	8 (0.67%)
11	UAE	8 (0.67%)
12	Kuwait	7 (0.58%)
13	Palestine	7 (0.58%)
14	Oman	5 (0.42%)
15	Canada	4 (0.33%)
16	Nepal	3 (0.25%)
17	Poland	3 (0.25%)
18	South Korea	3 (0.25%)
19	Thailand	3 (0.25%)
20	Tunisia	3 (0.25%)
21	Ireland	2 (0.17%)
22	South Africa	2 (0.17%)
23	USA	2 (0.17%)
24	Afghanistan	1 (0.08%)
25	Bahrein	1 (0.08%)
26	Cameroon	1 (0.08%)
27	China	1 (0.08%)
28	Greece	1 (0.08%)
29	Russia	1 (0.08%)
30	Spain	1 (0.08%)
31	Sudan	1 (0.08%)
32	West Indies	1 (0.08%)

## DISCUSSION & CONCLUSION

This study showed that the regular publication of each issue of Pakistan Journal of Medical Sciences got attention from the national and international research community relate to basic medical, biomedical, medicine & allied medical, surgery & allied surgical and radiological sciences with the average growth of publication maximum 59.8% to minimum 3.5% during the period of 2001 – 2010.

A critical analysis shows that submission from within the country are qite wide spread from all over Pakistan. However, as expected, most of the submissions from within Pakistan came from the city of Karachi followed by Lahore and then Rawalpindi-Islamabad. These are the major cities where research culture has progressed over the years. Similar is the case with other good quality peer reviewed journals published from Pakistan like Journal of Pakistan Medical Association which also has majority of local submissions from Karachi.

As regards submissions from overseas, it is also increasing every year. During the same period, maximum number of papers published from overseas were from Iran, Turkey, Nigeria and Saudi Arabia. However, it looks that in the coming years, submissions from other countries like Turkey and China will also increase manifold. All this gives strength with the result that Pakistan Journal of Medical Sciences has emerged as a leading peer reviewed biomedical journal in the region published from Pakistan attracting manuscripts in all the disciplines of medicine and related health sciences.

## References

[1] Smita SP, Vaishali SK, Research productive of faculties in sartmun sub-centre, Latur: A bibliometric study (2016). E-Library Science Research J.

[2] Ibrahim M, Jan SU (2015). Bibliometric analysis of the Journal of Pakistan Medical Association from 2009 to 2013. J Pak Med Assoc.

[3] Quinn N, Hensey O, McDowell DT (2013). A historical perspective of pediatric journals: a bibliometric analysis. Pediatrics.

[4] Sethi BB (2016). Periodical Literature Bibliometric Analysis: A case study of four International Journals. Library Philosophy and Practice (e-journal).

[5] Merigo JM, Nunez A, Influential journals in health research: a bibliometric study (2016). Globalization and Health.

[6] Chandrakar PP (2011). Indian LIS literature in international journals with specific reference to SSCI database: A bibliometric study. Library Philosophy and Practice e-journal.

[7] Sradkar PA, Daya DP (2016). International Research. Journal of Library & Information Science: Authorship Pattern and Degree of Collaboration in Academic Emergency Medicine.

[8] Chanda A, Chandra SS Authorship Trends and Collaborative Research in Veterinary Sciences: A Bibliometric Study. Chinese Librarianship: an International Electronic Journal 2012.

[9] Jawaid SA (2016). An introduction of PJMS. Pakistan Journal of Medical Sciences.

